# Computational Detection and Functional Analysis of Human Tissue-Specific A-to-I RNA Editing

**DOI:** 10.1371/journal.pone.0018129

**Published:** 2011-03-23

**Authors:** Tao He, Qiong Wang, Guihai Feng, Yaou Hu, Li Wang, Yumin Wang

**Affiliations:** 1 Beijing Institute of Biotechnology, Beijing, China; 2 Beijing Institute of Radiation Medicine, Beijing, China; University of New Orleans, United States of America

## Abstract

A-to-I RNA editing is a widespread post-transcriptional modification event in vertebrates. It could increase transcriptome and proteome diversity through recoding the genomic information and cross-linking other regulatory events, such as those mediated by alternative splicing, RNAi and microRNA (miRNA). Previous studies indicated that RNA editing can occur in a tissue-specific manner in response to the requirements of the local environment. We set out to systematically detect tissue-specific A-to-I RNA editing sites in 43 human tissues using bioinformatics approaches based on the Fisher's exact test and the Benjamini & Hochberg false discovery rate (FDR) multiple testing correction. Twenty-three sites in total were identified to be tissue-specific. One of them resulted in an altered amino acid residue which may prevent the phosphorylation of PARP-10 and affect its activity. Eight and two tissue-specific A-to-I RNA editing sites were predicted to destroy putative exonic splicing enhancers (ESEs) and exonic splicing silencers (ESSs), respectively. Brain-specific and ovary-specific A-to-I RNA editing sites were further verified by comparing the cDNA sequences with their corresponding genomic templates in multiple cell lines from brain, colon, breast, bone marrow, lymph, liver, ovary and kidney tissue. Our findings help to elucidate the role of A-to-I RNA editing in the regulation of tissue-specific development and function, and the approach utilized here can be broadened to study other types of tissue-specific substitution editing.

## Introduction

RNA editing is a widespread post-transcriptional modification mechanism that alters genetic information at the RNA level by nucleotide insertions, deletions or substitutions, which can contribute to the diversification of the transcriptome and proteome [1]–[2]. C-to-U substitutions and A-to-I substitutions are the two most common types of RNA editing. C-to-U substitution mostly exists in higher plant mitochondria and chloroplasts, and it is defined as the conversion of a single cytidine (C) base to a uridine (U) through deamination in primary transcripts [2]. A-to-I substitution, widely found in many vertebrates [3]–[8], is the modification by members of family of Adenosine Deaminases Acting on RNA (ADARs) of a single adenosine (A) base in primary transcripts to yield inosine (I). Since inosine is recognized as guanosine (G) by the splicing and translational machinery, A-to-I substitution leads to A-to-G transition in the edited substrate [9].

Nucleotide substitution of RNA editing can change the amino acid sequence, or create or destroy the translation initiation or termination codon. Nucleotide insertion or deletion from RNA editing can result in a translational frameshift that creates new open reading frames. The consequences of these editing events can increase the repertoire of available proteins [10]–[11]. Furtheremore, RNA editing can block the production of mature microRNA (miRNA) [12]–[14], redirect the miRNA to a new set of targets [15] and enrich the miRNA regulatory pathways. Dysregulation of the editing process may also contribute to the pathogenesis of certain diseases, such as dyschromatosis symmetrica hereditaria, acute myeloid leukemia and glioblastoma multiforme [16]–[18].

Previous studies have shown that some RNA editing events are tissue-specific and play important roles in physiological processes. More than 100 C-to-U substitutions in grape mitochondria were shown to be tissue-specific and may contribute to different tissue requirements [19]. A classic example of a C-to-U substitution occurs in the intestine-specific apolipoprotein in humans, creating a stop codon and a truncated apoliproprotein-B48 protein, which is less than half the size of the full-length apolipoprotein-B100 in the liver [20]. Anther-specific loss of atp6 RNA editing contributes to or causes cytoplasmic male sterility in *Sorghum bicolor* [21]. In another example, ovary/gut-specific U-to-C substitution and nerve cord/leg-specific A-to-I substitution of 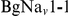 in cockroachs can generate tissue-specific functional variants of sodium channels with distinct gating properties [22]. Therefore, tissue-specific editing is thought to be required to modulate protein and non-coding RNA functionality in response to tissue-specific requirements. Systematic identification of tissue-specific RNA editing can help elucidate the molecular mechanisms of tissue development and function.

Although tens of thousands of A-to-I RNA editing events have been found in humans by computational and experimental methods, there is limited knowledge of its tissue-specificity in humans. To fully understand of this type of editing event, it is necessary to perform large-scale discovery and characterization of tissue-specific A-to-I RNA editing events. The methods based on expressed sequence tags (ESTs) for large-scale analysis of tissue specificity have been successfully used to study gene expression [23], alternative splicing [24]–[25] and alternative polyadenylation [26]. The vast collection of human ESTs and the associated annotations also provide an attractive opportunity to study tissue-specificity of A-to-I RNA editing. In this work, we demonstrated the effectiveness of a computational strategy by using ESTs and mRNA sequence data to detect tissue-specific A-to-I RNA editing in humans. Twenty-three A-to-I RNA editing sites were identified to be tissue-specific, one of which could alter the encoded amino acid and affect the protein function. Brain-specific and ovary-specific A-to-I RNA editing sites were further verified by comparing cDNA sequences with their corresponding genomic templates in several cell lines from brain, colon, breast, bone marrow, lymph, liver, ovary and kidney tissue. This strategy may be applied to study other types of tissue-specific substitution editing in different species.

## Results

### Computational detection of tissue-specific A-to-I RNA editing sites

Redundant records of the previously identified A-to-I RNA editing sites [3]–[5], [8] were removed and the unique sites were remapped to the assembled human genomic sequence. According to the alignment information downloaded from the UCSC genome browser website, all of the expressed sequences overlapping the same RNA editing site were grouped together and classified into two groups, edited or unedited, based on whether the nucleotide at the editing position is a guanosine (G) or adenine (A).

Following strict filters described in the methods section, the final tissue classification contained 379 cDNA libraries of 43 unique tissue types. For each tissue, the Fisher's exact test and the Benjamini & Hochberg false discovery rate (FDR) multiple testing correction were applied to detect the tissue-specific A-to-I RNA editing sites. We finally identified 23 tissue-specific A-to-I RNA editing sites in 13 different tissues (Table 1). The top four distributions were tonsil, adipose tissue, pancreas and nerve, which contained 8, 2, 2 and 2 sites, respectively. Other tissues containing only one observed tissue-specific event were trachea, thyroid, salivary gland, pituitary gland, ovary, ear, connective tissue, brain and blood.

**Table 1:** Tissue-specific A-to-I RNA editing sites by computational detection.

Tissue	Tissue-specific A-to-I RNA editing site			FDR corrected *P* value	Gene related information
	Chromosome	Strand	Position		
adipose tissue	chr8	-	117738703	0.000197	EIF3H
	chr5	+	150017483	0.007937	SYNPO
tonsil	chr6	+	52466294	0.002747	EFHC1
	chr6	+	52466305	0.002747	
	chr6	+	52466312	0.002747	
	chr6	+	52466320	0.002747	
	chr6	+	52466321	0.002747	
	chr6	+	52466350	0.002747	
	chr6	+	52466400	0.002747	
	chr6	+	52466401	0.002747	
trachea	chr3	-	150569793	0.000233	TM4SF1
thyroid	chr8	-	11737640	0.001768	CTSB
salivary gland	chr15	+	39384948	0.010084	
pituitary gland	chr4	+	57021844	0.015649	PAICS
pancreas	chr15	-	40622466	0.000206	LRRC57
	chr15	-	40622469	0.000206	
ovary	chrX	-	128767292	7.79e-008	ZDHHC9
nerve	chr8	-	143850023	0.000153	LYNX1
	chr14	-	105401091	0.000609	
ear	chr5	-	81607256	0.000206	RPS23
connective tissue	chr10	-	44192920	0.001107	CXCL12
brain	chr4	+	57021835	5.43e-006	PAICS
blood	chr8	-	145130527	0.001287	PARP10

An RNA editing event happens after gene transcription. Therefore, the expression profile of a gene limits the possibility of an RNA editing event. To test whether high expression of a gene in a tissue could increase its RNA editing level in the same tissue or not, we investigated the tissue-preferred expression of genes which contain tissue-specific RNA editing sites. By searching the Tissue-specific Gene Expression and Regulation (TiGER) database, we found that the CXCL12 gene with a connective-tissue-specific A-to-I RNA editing site in its 3′-UTR (or intron in other isoforms) was preferentially expressed in soft tissue, heart and spleen. CXCL12 can activate lymphocytes and take part in the metastasis of prostate cancer [27]. Connective tissue is the main component of soft tissue, and high expression of CXCL12 in soft tissue may increase its RNA editing level in the connective tissue. However, the vast majority of genes observed here with the tissue-specific A-to-I RNA editing sites did not show the same tissue-specificity in their gene expression profiles.

On the other hand, we analyzed the tissue-preferred expression of all annotated 2,040 genes with 18,616 A-to-I RNA editing sites. Three hundred and seventy-eight of these genes were expressed in a tissue-specific manner according to the TiGER database collection. Except CXCL12 as mentioned above, there was only one muscle-specific gene, SYNPO (an actin-associated protein), with an adipose-tissue-specific A-to-I RNA editing site. This observation indicated that the vast majority of tissue-preferred genes in this study did not contain putative tissue-specific A-to-I RNA editing sites.

Therefore, we concluded that the A-to-I RNA editing and the expression of the corresponding editing substrate did not show the same tissue preferences in our study. That is, high expression of a gene does not increase its RNA editing level, and the tissue-specific editing can exist in transcripts that are widely expressed.

### Experimental verification of brain-specific and ovary-specific RNA editing sites

**Figure 1:**
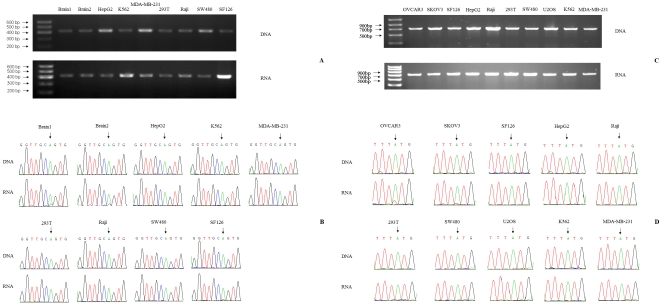
Experimental validation of the predicted brain-specific and ovary-specific RNA editing sites. (A) The up/downstream region of the brain-specific RNA editing site was amplified successfully from cDNAs and gDNA of two adjacent non-cancerous brain tissues, as well as the HepG2, K562, MDA-MB-231, 293T, Raji, SW480 and SF126 cell lines. (B) Sequencing results of paired genomic DNA (control) and cDNA from the same human brain specimens and seven human cell lines. A mixed peak of A and G in the cDNA sample but not in the genomic counterpart indicates the presence of RNA editing in both adjacent non-cancerous brain tissues and the human glioma cell line SF126. (C) The up/downstream region of the ovary-specific RNA editing site was amplified successfully from cDNAs and gDNA of the OVCAR3, SKOV3, SF126, HepG2, Raji, 293T, SW480, U2OS, K562 and MDA-MB-231 cell lines. (D) Sequencing results of paired genomic DNA (control) and cDNA from the same ten human cell lines. A mixed peak of A and G in the cDNA sample but not in the genomic counterpart indicates the presence of RNA editing in the two human ovarian cancer cell lines SKOV3 and OVCAR3.

To experimentally validate the predicted brain-specific and ovary-specific editing sites, two human tissue samples (brain) and ten human cell lines (from brain, ovary, colon, breast, bone, bone marrow, lymph, liver, and kidney) were used. We sequenced matching DNA and RNA samples retrieved from the same specimen. As shown in Figure 1a and 1c, the edited substrates were amplified successfully in all tissue samples and cell lines. The absence of visible bands in the no-RT controls confirmed that there was no DNA contamination in RNA used to generate the cDNA. The PCR products were sequenced as a population without cloning. When the PCR products were directly sequenced, editing was determined by the presence of an unambiguous trace of guanosine in positions for which the genomic DNA clearly indicated the presence of an adenosine. We verified the predicted brain-specific editing events in both the brain tissue samples and the human glioma cell line SF126 (Figure 1b) and the predicted ovary-specific editing events in two human ovarian cancer cell lines (SKOV3 and OVCAR3, Figure 1d). The editing level was represented as a percentage estimated from the ratio of the ‘G’ peak over the sum of the ‘G’ and ‘A’ peaks in the sequencing chromatogram. The estimated editing level of brain-specific RNA editing was 17.7% (151/855) in the Brain1 tissue sample, 22.6% (240/1061) in the Brain2 tissue sample and 10.1% (85/842) in the glioma cell line SF126. No corresponding editing events were observed in the other 6 cell lines from colon, breast, bone marrow, lymph, liver and kidney (Figure 1b). The positive experimental results obtained only in the brain tissue and cell line indicated that the A-to-I RNA editing event that occurred at site chr4_+_ 57021835 was brain-specific. The estimated editing level of ovary-specific RNA editing was 16.8% (171/1015) in the human ovarian cancer cell line OVCAR3 and 7.9% (70/888) in the human ovarian cancer cell line SKOV3. No corresponding editing events were observed in the other 8 cell lines from brain, colon, breast, bone, bone marrow, lymph, liver, and kidney (Figure 1d). The positive experimental results obtained only in the two human ovarian cancer cell lines indicated that the A-to-I RNA editing event which occurred at site chrX_−_128767292 was ovary-specific.

### Tissue-specific RNA editing sites in protein coding regions

Some A-to-I RNA editing sites are located in protein-coding regions, whereas the majority is found in non-coding regions. An editing site within the protein-coding region of an mRNA can result in a sequence change that may lead to an amino acid alteration in the protein. By analysis using EditFunc, one blood tissue-specific RNA editing site was found to alter an amino acid residue. The editing site mapped to chr8_−_145130527 changes the serine residue at position 507 of the PARP-10 protein (Genbank accession: NP_116178) to a glycine residue, which was predicted as a putative phosphorylation site by the EditFunc web server with the use of the NetPhos software [28]. Phosphorylation of a serine, a threonine or a tyrosine residue is one of the most common mechanisms of regulating protein function. Therefore, this blood-specific editing event may prevent the phosphorylation of PARP-10 and alter its activity.

PARP-10 belongs to the family of Poly (ADP-ribose) polymerases, which regulates gene transcription by altering chromatin organization by adding ADP-ribose to histones. PARP-10 was reported to interact with the Myc protein and inhibit cell proliferation [29]. From its tissue expression pattern, PARP-10 is preferentially expressed in hematopoietic tissues, although it can be detected in 16 different tissue types [29]. The blood-specific editing of PARP-10 showed a similar preference in its expression profile, implying that the blood-specific editing may be involved in the control of cell proliferation in hematopoietic tissues.

### Tissue-specific RNA editing sites in exonic splicing enhancers (ESEs) and exonic splicing silencers (ESSs)

In recent years, some evidences have accumulated showing that splicing and editing can influence each other [30]–[33]. To investigate whether the tissue-specific A-to-I editing may disrupt the functional elements of ESE and ESS, we analyzed the edited and unedited exon sequences with the EditFunc web server using the programs ESEfinder [34]–[35] and FAS-ESS [36]. Eight tissue-specific A-to-I editing sites were predicted to alter the SF2/ASF, SC35 and SRp40 ESE motifs (Table S1), and two tissue-specific A-to-I RNA editing sites were predicted to change four ESS hexamers (GGGAGG, TAGGTA, TTAGGT and CTTAGG, Table S2). It has been shown that the mutation of an ESE or ESS sequence can inactivate its function and affect pre-mRNA splicing [37]–[38]. Therefore, these tissue-specific A-to-I RNA editing sites may disrupt ESEs or ESSs and lead to changes in transcript splicing patterns.

## Discussion

RNA editing is an important post-transcriptional regulation that can increase protein diversity and enrich the regulation of non-coding RNA. Although a few studies have indicated that RNA editing is an indispensable modulation in response to the requirements of specific cell types, it has been a challenge to gain an overview of the global landscape of tissue-specific editing. In this study, we successfully detected human tissue-specific A-to-I editing sites by statistically analyzing EST/mRNA sequences. The overwhelming majority of the known RNA editing sites used here was found in the non-coding sequences, and most of the predicted editing sites were located in the non-coding regions as well. By gaining a deeper understanding of the non-coding sequences, we should begin to know more about the functions of the tissue-specific RNA editing.

Interestingly, most of the genes containing the tissue-specific A-to-I RNA editing did not exhibit tissue-specific expression. On the contrary, many tissue-specific genes were not discovered to have the predicted tissue-specific A-to-I RNA editing sites, although we could not exclude the possibility that they may have other unknown tissue-specific RNA editing sites. This implies that the tissue-specific editing event is a modulatory mechanism required for tissue-specific development but that its role is independent of the regulation of tissue-specific gene expression. The members of the family of ADARs are the only enzymes that are known to regulate A-to-I RNA editing levels. However, it seems that the regulation by ADARs cannot completely explain how tissue-specific editing occurs. Recent studies indicated that editing levels can increase or decrease with a constant (or not significantly changed) protein expression of ADARs [39]–[40], consistent with the opinion of Jacobs and colleagues that the differences in editing patterns may not be mediated solely by ADAR expression levels [41]. Take together, these observations indicate that there may be factors in addition to the ADARs that are involved in the tissue-specific A-to-I RNA editing process.

RNA-seq data can also be used to detect tissue-specific editing if the read sequences are treated as EST/mRNA sequences. However, the high expense of whole genome and transcriptome sequencing currently restricts its application for RNA editing analysis, and there are only three published works that have utilized high-throughput sequencing to detect RNA editing at present [8] [19] [39]. Furthermore, the application of whole genome and transcriptome sequencing for detection of the human tissue-specific A-to-I RNA editing would be even more costly. For each individual, whole genome sequencing should be performed once or twice (replicate), and whole transcriptome sequencing should be performed in each tissue. For studies involving different donors, whole genome and transcriptome sequencing would be required for each donor and their tissues, significantly adding to the overall cost and labor requirements. However, with the development of lower cost next generation sequencing technology, significantly more data may be accumulated, and it is expected that more reliable and novel observations will be realized by using this approach.

Finally, we have to note that there are probably many more tissue-specific editing sites than those identified in this work for the following reasons. (i) The coverage of expressed sequences in the same editing sites in all tissues are not equivalent. Therefore, many editing sites may be detected in a only few tissues but not in others where there are just too few or no expressed sequences. (ii) The Fisher's exact test with the Benjamini & Hochberg correction is usually considered strict and may cause us to miss detection of some true tissue-specific editing sites. (iii) Finally, many A-to-I RNA editing sites have been uncovered to date, and the 23 tissue-specific A-to-I RNA editing sites predicted here still represents a small portion of the actual tissue-specific RNA editing repertoire. Nevertheless, this is the first study to explore tissue-specific A-to-I RNA editing in humans, and the information gained here may facilitate the understanding of regulation by RNA editing related to the unique functions of tissues.

## Materials and Methods

### Data sources

Five sources of data were required for our analysis, including known A-to-I RNA editing sites, the human reference genomic sequences, the human mRNA/EST sequences, the alignments between the human mRNA/EST and reference genome sequences, and the human mRNA/EST library information. The total of 32,316 non-redundant A-to-I RNA editing sites identified by different methods were collected from four published works [3]–[5] [8]. The other four resources, such as the human reference genomic sequences (hg18), the mRNA/EST sequences, the ‘gbCdnaInfo.txt’ flat file (alignment between the human mRNAs/ESTs and genome sequences), and the ‘tissue.txt’ flat file (human mRNA/EST library information) were all downloaded from the UCSC genome browser website [42]. First, all of the known editing sites were remapped to the human genome sequences (hg18). Subsequently, the expressed sequences of mRNAs/ESTs overlapping the same RNA editing site were grouped together based on the alignment information. Every grouped mRNA or EST sequence was classified as edited or unedited according to whether the nucleotide at the position of the known editing is a guanosine (G) or adenine (A).

### Tissue classification

Four hundred and ninety cDNA libraries with tissue annotations were downloaded from the UCSC website. A total of 111 cDNA libraries were excluded from the original set because these libraries lacked clear tissue source information or were from mixed tissue samples. Furthermore, libraries recorded as having the same tissue source (e.g. ‘brain’) were combined into a single category, including both normal and cancerous samples from the same tissue. Finally, we filtered and grouped 379 cDNA libraries into 43 unique tissue types (Table 2).

**Table 2:** Distribution of mRNA/EST sequences and cDNA libraries identified with A-to-I editing sites among 43 tissue types.

Tissue	No. of libraries	No. of mRNAs/ESTs
adipose tissue	5	68
adrenal gland	5	120
ascites	1	76
bladder	2	22
blood	10	139
bone	12	385
bone marrow	10	101
brain	52	3090
cervix	6	127
connective tissue	6	161
ear	1	17
esophagus	3	23
eye	22	583
heart	2	25
intestine	20	366
kidney	13	336
larynx	3	4
liver	10	473
lung	16	438
lymph	19	429
lymph node	2	187
mammary gland	11	271
mouth	7	148
muscle	6	233
nerve	23	651
ovary	8	169
pancreas	10	707
parathyroid	1	62
pharynx	1	41
pituitary gland	3	47
placenta	9	566
prostate	11	265
salivary gland	3	62
skin	23	488
spleen	2	271
stomach	4	78
testis	6	386
thymus	1	520
thyroid	4	79
tonsil	4	292
trachea	1	200
uterus	18	506
vascular	3	13

### Determination of tissue specificity

As a measure of tissue-specificity, Fisher's exact test was applied to assess the significance of different RNA editing levels in all tissues, and the Benjamini-Hochberg method was used to estimate the total FDR in each tissue for correction of multiple testing.

For each RNA editing site 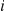, 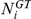 and 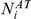 represent the total numbers of ESTs/mRNAs in tissue T observed in edited form or unedited form, respectively. Similarly, 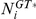 and 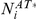 are the total numbers of ESTs/mRNAs in the pool of all other tissues observed in edited form or unedited form, respectively. The Fisher's exact test was used to compute the
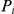
value from any 2 by 2 table.

The following simple procedure to control the FDR at level 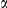 was proposed by Benjamini and Hochberg [43]. For m tests in tissue T, the *P* values were ranked in ascending order 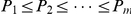 and the null hypothesis corresponding to 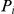 was denoted by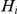. The 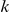 variable represented the largest 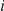 for which 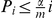 and all null hypotheses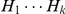 were rejected. In other words, each 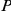 value (starting with the highest) was checked for this requirement; at the first *P* value that met the requirement, its corresponding null hypothesis and all those having smaller 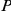
values were rejected. The desired confidence level was 0.95 (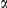 = 0.05).

### Expression profiles of tissue-specific genes

To explore whether genes containing the A-to-I RNA editing sites were expressed in a tissue specific manner or not, we searched their expression profiles from the TiGER database [44]. This database contains a collection of 7,261 tissue-specific genes from 30 tissues based on the expression enrichment (EE) values and statistical significance.

### Clinical samples and cell lines

Two brain adjacent non-cancerous tissue samples and ten cell lines were used in this study for experimental validation. The brain tissue samples were obtained from the 307 Hospital of PLA with the written informed consent of patients and with approval for experiments from the ethics committees of the hospital and the Beijing Institute of Biotechnology. The human glioma cell line SF126 was purchased from the Cancer Institute and Hospital, Chinese Academy of Medical Sciences (CAMS). The two human ovarian cancer cell lines (SKOV3 and OVCAR3) were supplied by Peng Peng (Peking Union Medical College Hospital, Beijing, China). The human colon cancer cell line SW480, the human estrogen receptor (ER)-negative breast cancer cell line MDA-MB-231 and the human osteosarcoma cell line U2OS were supplied by Xuemin Zhang (National Center of Biomedical Analysis, Beijing, China). The human chronic myeloid leukemia cell line K562 and the human Burkitt's lymphoma cell line Raji were gifts from Qingfeng Du (Nanfang Hospital, Southern Medical University, Guangzhou, China). The human renal epithelial cell line 293T and the human hepatoma cell line HepG2 were supplied by Yan Lin (Beijing Institute of Biotechnology, Beijing, China).

### Cell culture

K562, SW480, SKOV3 and Raji cell lines were cultured in RPMI 1640 media (Gibco) supplemented with 10% fetal bovine serum (FBS, Gibco), 100 U/ml penicillin and 0.1 mg/ml streptomycin (Hyclone). HepG2, MDA-MB-231, OVCAR3, U2OS and 293T cell lines were grown in Dulbecco's Modified Eagle Medium (DMEM) (Gibco) with 10% FBS, 100 U/ml penicillin and 0.1 mg/ml streptomycin. SF126 was maintained in Minimum Essential Medium (MEM) (Gibco) with 10% FBS, 100 U/ml penicillin and 0.1 mg/ml streptomycin. All cells were cultured at 37°C in a 5% CO_2_ incubator with humidified air.

### RNA extraction and RT-PCR

For experimental validation of brain and an ovary tissue-specific RNA editing sites, total RNA and gDNA of two brain tissue samples isolated from the same specimen and ten cell lines were processed using standard protocols for reverse transcription and PCR. To remove genomic DNA contamination, RNA samples were treated with DNase I (Takara, Otsu, Shiga, Japan). First-strand cDNA was synthesized from the total RNA with the Transcriptor High fidelity cDNA Synthesis Kit (Roche) using random primers. Using the cDNA and gDNA as templates, PCR was performed according to standard procedures with 30 pM of each primer and 2.5 U rTaq DNA polymerase (Takara, Otsu, Shiga, Japan) to amplify the edited transcripts and the genomic DNA. The cycling conditions for amplification were as follows: initial denaturation at 95°C for 5 min, then 30 cycles at 95°C for 30 s, 59°C for 30 s, and 72°C for 30 s, followed by a final extension at 72°C for 10 min. Control experiments were conducted without the reverse transcriptase enzyme added (no RT control) to verify that the amplified products were from the reverse transcribed mRNA and not from contaminating genomic DNA. The products were resolved by electrophoresis on a 1% w/v agarose gel in TAE buffer (40 mmol/L Tris-acetate, 2 mmol/L Na2EDTA, 2H2O) and stained with ethidium bromide. Finally, DNA bands were quantified using a Gel Imaging Analysis System BINTA 2020D and the GelPro32 software (Beijing BINTA Instrument Technology Co., Ltd., China). The primers were synthesized by Beijing AuGCT Biotechnology Co., Ltd, and sequencing of PCR products was performed by Beijing Tianyi Huiyuan Life Science & Technology, Inc. The following primers were used to detect the genomic DNA and mRNA:

BR-L: 5′-TggTTCTTgggTTCTCCCgAAgCCT-3′,

BR-R: 5′-AggTACCAATgTgTggCAgTCCA-3′,

OV-L: 5′- AAATCCTCCCAAgCTgCTgCACg-3′,

OV-R: 5′- AgTgCTgggCTTTCCCTCACTCA-3′.

### Predicting the functional effects of tissue-specific RNA editing sites

EditFunc (http://www.compbio.net.cn/editfunc), a web server for predicting potential effects of RNA substitution editing, was used to predict the functional effects of the tissue-specific RNA editing sites. EditFunc can predict the effects of the RNA editing sites at the transcriptional level, including changes in canonical splice site sequences, exonic splicing enhancers, exonic splicing silencers, Piwi-interacting RNAs (piRNAs) and miRNAs compared with their targets. It can also predict the effects of RNA editing sites at the translational level, including alterations in the initiation codon, termination codon, amino acid residues, physicochemical properties, glycosylatioin sites, phosphorylation sites, propeptide cleavage sites and signal peptide domains.

According to the annotated piRNA [45], miRNA [46] and the corresponding target datasets [47], five EditFunc prediction options for piRNA, miRNA target, precursor miRNA, mature miRNA and miRNA seed allow the user to detect whether the queried editing site is located at non-coding RNAs and their functional regions or not. The splice sites, translational initiation and termination codons were detected in the genome by the GeneID program [48], and the results were used to identify whether the RNA editing site may damage the normal mRNA splicing or protein translation processes.

The putative ESSs were scanned in all exon sequences of human genes by using the FAS-hex-3 set [36]. RNA editing sites located at these ESSs were cataloged as potential sites that could disturb the silencer activity. EditFunc was also used to scan exon sequences based on previously published nucleotide-frequency matrices [35] to identify putative ESEs responsive to the human serine/arginine-rich proteins (SR proteins) SF2/ASF, SC35, SRp40 and SRp5. ESEs with scores over the threshold [35] were regarded as the functional elements in this study. If the RNA editing site reduced the score of the ESE to below threshold value, it was annotated as a potential site that could disrupt activity at this ESE.

Six EditFunc prediction options for propeptide cleavage site, signal peptide, N-linked glycosylatioin, O-linked glycosylatioin, C-linked glycosylatioin and Phosphorylation were used to first execute external programs Prop [49], Signalp [50], netNglyc (http://www.cbs.dtu.dk/services/NetNGlyc/), netOglyc [51], netCglyc[52] and Netphos [28], and then to map the RNA editing sites to these protein functional sites or domains and to assess their potential effects on normal protein processing or post-translational modification.

## Supporting Information

Table S1 (DOC)

Table S2 (DOC)
