# Supplementary material for: Computational Detection and Functional Analysis of Human Tissue-Specific A-to-I RNA Editing
Source: PLoS One. 2011 Mar 23;6(3):e18129. doi: 10.1371/journal.pone.0018129 (PMC3063316; doi:10.1371/journal.pone.0018129)
Supplement: Table S1 — (DOC) [file pone.0018129.s001.doc]

Supplement Materials

Table S1 Eight tissue-specific A-to-I editing sites predicted to destroy the SF2/ASF, SC35 and SRp40 ESE motifs

| **Editing Site** | | | **Gene ID** | **Gene**  **Name** | **GenBank ID** | **Exon Start** | **Exon End** | **Protein Name** | **ESE Start** | **Seq1** | **Score1** | **Seq2** | **Score2** | **Effect** |
| --- | --- | --- | --- | --- | --- | --- | --- | --- | --- | --- | --- | --- | --- | --- |
| **Chr** | **Strand** | **Loci** |
| chr8 | - | 117738703 | 8667 | EIF3H | NM_003756 | 117738634 | 117738734 | SF2/ASF  (lgM-BRCA1) | 117738700 | CTCACTA | 3.43 | CTCGCTA | 1.71 | lose |
| chr8 | - | 117738703 | 8667 | EIF3H | NM_003756 | 117738634 | 117738734 | SRp40 | 117738699 | TCACTAA | 3.13 | TCGCTAA | 0.52 | lose |
| chr8 | - | 117738703 | 8667 | EIF3H | NM_003756 | 117738634 | 117738734 | SF2/ASF | 117738700 | CTCACTA | 3.35 | CTCGCTA | 0.45 | lose |
| chr5 | + | 150017483 | 11346 | SYNPO | NM_007286 | 150016158 | 150018985 | SRp40 | 150017479 | TCACAAT | 3.38 | TCACGAT | 0.83 | lose |
| chr6 | + | 52466294 | 114327 | EFHC1 | NM_018100 | 52465026 | 52468542 | SC35 | 52466288 | AGTCCCAG | 3.1 | AGTCCCGG | 1.8 | lose |
| chr6 | + | 52466294 | 114327 | EFHC1 | NM_018100 | 52465026 | 52468542 | SRp40 | 52466290 | TCCCAGC | 3.08 | TCCCGGC | 0.53 | lose |
| chr6 | + | 52466305 | 114327 | EFHC1 | NM_018100 | 52465026 | 52468542 | SF2/ASF | 52466299 | CTCGGGA | 1.96 | CTCGGGG | 1.23 | lose |
| chr6 | + | 52466312 | 114327 | EFHC1 | NM_018100 | 52465026 | 52468542 | SC35 | 52466306 | GGCTTAAA | 2.71 | GGCTTAGA | 1.41 | lose |
| chr6 | + | 52466401 | 114327 | EFHC1 | NM_018100 | 52465026 | 52468542 | SRp40 | 52466401 | AGACTCC | 3.05 | GGACTCC | 1.6 | lose |
| chr8 | - | 143850023 | 66004 | LYNX1 | NM_177477 | 143849616 | 143853783 | SF2/ASF | 143850020 | CGCAGGG | 4.8 | CGCGGGG | 1.9 | lose |
| chr8 | - | 143850023 | 66004 | LYNX1 | NM_177477 | 143849616 | 143853783 | SRp40 | 143850021 | TCGCAGG | 3.72 | TCGCGGG | 1.17 | lose |
| chr5 | - | 81607256 | 6228 | RPS23 | NM_001025 | 81604894 | 81607830 | SF2/ASF | 81607253 | CTTAGGT | 2.2 | CTTGGGT | -0.7 | lose |
| chr5 | - | 81607256 | 6228 | RPS23 | NM_001025 | 81604894 | 81607830 | SF2/ASF(lgM-BRCA1) | 81607253 | CTTAGGT | 2.47 | CTTGGGT | 0.75 | lose |
| chr5 | - | 81607256 | 6228 | RPS23 | NM_001025 | 81604894 | 81607830 | SRp40 | 81607254 | TCTTAGG | 2.9 | TCTTGGG | 0.35 | lose |

**Note:** ‘Chr’= the chromosome. ‘Strand’= the transcription direction of editing substrate. ‘Loci’= the chromosome location of the editing site. Gene IDs= the NCBI Entrez Gene IDs. ‘Exon Start’= the start position of the exon with the putative ESE in human chromosome; ‘Exon End’ = the end position of the exon with the putative ESE in human chromosome; ‘Protein Name’= the name of human SR proteins which the putative ESEs response to. ‘Seq1’= the un-edited ESE sequence; ‘Seq2’= the edited ESE sequence. ‘Score1’ = the scores of the un-edited ESE sequences calculated based on the nucleotide-frequency matrices; ‘Score2’= the scores of the edited ESE sequences calculated based on the nucleotide-frequency matrices. ‘Effect’ = the editing effect on ESEs; “Lose” = the functional loss of ESE.
