# Supplementary material for: Computational Detection and Functional Analysis of Human Tissue-Specific A-to-I RNA Editing
Source: PLoS One. 2011 Mar 23;6(3):e18129. doi: 10.1371/journal.pone.0018129 (PMC3063316; doi:10.1371/journal.pone.0018129)
Supplement: Table S2 — (DOC) [file pone.0018129.s002.doc]

Table S2 Two tissue-specific A-to-I RNA editing sites predicted to destroy the four ESS hexamers

| **Editing Site** | | | **Gene ID** | **Gene Name** | **GenBank ID** | **Exon Start** | **Exon End** | **ESS Start** | **Motif** | **Editing Type** |
| --- | --- | --- | --- | --- | --- | --- | --- | --- | --- | --- |
| **Chr** | **Strand** | **Loci** |
| chr6 | + | 52466305 | 114327 | EFHC1 | NM_018100 | 52465026 | 52468542 | 52466302 | GGGAGG | A->G |
| chr5 | - | 81607256 | 6228 | RPS23 | NM_001025 | 81604894 | 81607830 | 81607252 | TAGGTA | A->G |
| chr5 | - | 81607256 | 6228 | RPS23 | NM_001025 | 81604894 | 81607830 | 81607253 | TTAGGT | A->G |
| chr5 | - | 81607256 | 6228 | RPS23 | NM_001025 | 81604894 | 81607830 | 81607254 | CTTAGG | A->G |

**Note**: ‘‘Chr’= the chromosome. ‘Strand’= the transcription direction of editing substrate. ‘Loci’= the chromosome location of the editing site. Gene IDs= the NCBI Entrez Gene IDs. ‘Exon Start’= the start position of the exon with the putative ESE in human chromosome; ‘Exon End’ = the end position of the exon with the putative ESE in human chromosome;’Motif’ = the hexamers identified as ESS candidates.
